# Advancements in cell-penetrating monoclonal antibody treatment

**DOI:** 10.18632/oncotarget.28674

**Published:** 2024-11-22

**Authors:** Sai Pallavi Pradeep, Raman Bahal

**Keywords:** monoclonal anti-bodies, cell penetration, nucleic acid delivery, 3E10

***News on**: Next-generation cell-penetrating antibodies for tumor targeting and RAD51 inhibition by Rackear et al. Oncotarget. 2024; 15:699–713. https://doi.org/10.18632/oncotarget.28651. [PubMed]*

Monoclonal antibodies (mAb) have emerged as a promising tool in advancing personalized medicine. In 1984, the first approved monoclonal antibody therapy (orthoclone OKT3) was used to prevent kidney transplant rejections. OKT3 was a murine mAb targeting T-cell-expressed CD3 and was discontinued in 2011 due to its efficacy and immunogenic potential [1]. Researchers focused on improving the long-term therapeutic use of antibodies by developing techniques to humanize rodent antibodies. Humanized antibodies were generated using the complementary-determining region grafting technique, where the non-human variable region is transplanted with human framework regions. The first humanized mAb against the IL-2 receptor was approved in 1997 to prevent transplant rejection [1]. Fully human mAbs are now developed using phage display technology, mouse hybridoma, and B-cell technology. These techniques have made it possible to clinically utilize mAbs against diseases like cancer and autoimmune diseases. mAbs have transformed cancer treatment by offering targeted therapy with fewer side effects than conventional chemotherapy. They have been used as checkpoint immune therapies targeting programmed death-ligand, programmed death-ligand 1, or cytotoxic T lymphocyte-associated antigen 4. They have also been used as antibody-drug conjugates, combining the mAb potential for targeting overexpressed receptors on cancer cells with a cytotoxic payload (tubulin inhibitors, DNA-damaging agents, immunomodulators) [2]. mAb conjugates have been successful in targeting cell surface antigens. However, a significant fraction of therapeutic targets is intracellular. Intracellular transport of mAbs is limited due to the entrapment within the compartments of the endocytosis pathway. mAbs are usually degraded in the lysosome or are recycled through neonatal Fc receptors. Researchers have employed cell-penetrating peptides, polymer-based materials (polyethylene glycol), and liposomes to encapsulate and deliver mAbs [3].

A more recent development is the use of systemic lupus erythematosus (SLE)-derived auto-antibodies, such as 3E10, that escape endosomal entrapment. 3E10 has an affinity for nucleic acids and can penetrate the plasma and nuclear membrane. It undergoes uptake via ENT2 nucleoside transporter, which is present on both barriers. ENT2 is also overexpressed in many cancers and hence can be used for tumor targeting and intracellular delivery [4]. 3E10 was found to bind to RAD51 and can potentially be used in tumors with defective DNA repair pathways like BRCA2 and PTEN deficiencies [5]. 3E10 has been humanized to reduce immunogenicity and improve pharmacokinetic properties in humans [6]. Several of the humanized 3E10 variants were observed to retain RAD51 binding properties. RAD51 binding can lead to inhibition of homologous directed repair and replication fork collapse in cells deficient in DNA damage repair. The humanized 3E10 variants have also been explored for the delivery of nucleic acids like mRNA. GFP mRNA expression was shown to differ across 3E10 variants. 3E10 variants having lower binding affinity to GFP mRNA showed faster release, and maximum expression. [6]. The humanized variants of 3E10 have varying affinity to nucleic acids which can be explored to optimize these antibodies for different therapeutic applications.

3E10 represents a new class of mAbs that can deliver diverse payloads such as mRNA, other nucleic acids or cytotoxic compounds to tumors. 3E10 can be evaluated for its binding affinity to antisense oligonucleotides and the delivery of these constructs to degrade target mRNA. The targeting potential of E310 can also be explored for the delivery of other constructs like chemically modified peptide nucleic acids (PNA), locked nucleic acids (LNAs) [7] and other class of antisense nucleic acids which can bind to mRNA inhibiting translation [8]. PNAs and antisense oligonucleotides can be conjugated by cleavable linkages, to target oncogenes [9] and RAD51 simultaneously. Similarly, 3E10 antibodies can be used to deliver next generation-PNAs to target genomic DNA during *in vivo* studies [9]. The combination of tumor targeting, nucleic acid delivery, and RAD51 inhibition positions 3E10 as a promising candidate for further development in cancer treatment, gene therapy, and other innovative treatment strategies.
